# Age at menarche and risk of hypertension in Chinese adult women: Results from a large representative nationwide population

**DOI:** 10.1111/jch.14321

**Published:** 2021-07-13

**Authors:** Lu Chen, Linfeng Zhang, Zuo Chen, Xin Wang, Congyi Zheng, Yuting Kang, Haoqi Zhou, Zengwu Wang, Runlin Gao

**Affiliations:** ^1^ Division of Prevention and Community Health National Center for Cardiovascular Disease Fuwai Hospital, Peking Union Medical College & Chinese Academy of Medical Sciences Mentougou District Beijing China; ^2^ Department of Cardiology Fuwai Hospital, Peking Union Medical College & Chinese Academy of Medical Sciences Xicheng District Beijing China

**Keywords:** age at menarche, Chinese women, hypertension

## Abstract

This study explored the association between age at menarche and the risk of hypertension in Chinese women. A total of 234 867 women aged ≥18 years from the China Hypertension Survey were included in this study. Participants were required to complete a standard questionnaire. Blood pressure and physical examination of the participants were performed by trained medical staff. Spearman correlation analysis was used to explore the correlation between age at menarche and other individual characteristics. Logistic regression was used to estimate the odds ratios for hypertension by age in years at menarche. The average age at menarche in Chinese women was 14.8 years. Women who were older at menarche were more likely to have a higher body mass index, larger waist circumference, smoke, and have a primary education (*p *< .05). After adjustments, odds ratios (95% confidence interval) for hypertension across age at menarche groups were 0.912 (0.877–0.948), 0.927 (0.893–0.963), 1.00 (reference), 1.061 (1.020–1.102), and 1.129 (1.090–1.169) for those aged ≤13, 14, 15 (reference), 16, and ≥17 years at menarche, respectively. Each 1‐year delay in menarche was associated with a 6.2% increase in the prevalence of hypertension (odds ratio, 1.062; 95% confidence interval, 1.053–1.071). The positive association between age at menarche and hypertension was evident among age at recruitment groups, BMI categories, and education levels. This association was stronger in urban women and postmenopausal women. Our findings suggest that late menarche is related to a higher risk of hypertension among Chinese adult women, and this association appeared similar among different subgroups.

## 1 INTRODUCTION

Hypertension is an important cause of cardiovascular disease and death and has been recognized as a public health problem worldwide.^1^, ^2^ In China, cardiovascular disease is the leading cause of death, accounting for approximately 45% of all deaths.^3^ The China Hypertension Survey reported that 23.2% (estimated 244.5 million) of Chinese adults aged ≥18 years have hypertension, and another 41.3% (estimated 435.3 million) have pre‐hypertension.^4^ Menarche is the first menstruation of a girl, and it is a marker of puberty and the onset of ovarian and other endocrine functions related to reproduction.^5^ Age at menarche is associated with the risk of cardiovascular disease,^6^, ^7^, ^8^ type 2 diabetes,^9^, ^10^ and obesity.^11^, ^12^

The results of previous studies on the relationship between age at menarche and the risk of hypertension were inconsistent. A study on women in Brazil reported that age at menarche negatively correlated with hypertension.^13^ A study on 1423 low‐income Bangladeshi women found no significant association between age at menarche and hypertension.^14^ Canoy found a U‐shaped relationship between age at menarche and hypertension in the British female population.^15^ Gang Liu found that age at menarche was positively associated with hypertension among women in a city in southwestern China.^16^ The reasons for this difference may include genetic background, birth weight, mean age at menarche, living environment, and lifestyle behavior among different populations.^17^, ^18^, ^19^

At present, there is a lack of nationally representative large‐sample studies to explore the relationship between age at menarche and hypertension in Chinese women. Based on the China Hypertension Survey, which is representative of the whole country, we investigated the relationship between age at menarche and hypertension and examined the relationship by subgroups among Chinese women.

## 2 METHODS

### 2.1 Study participants

**FIGURE 1:**
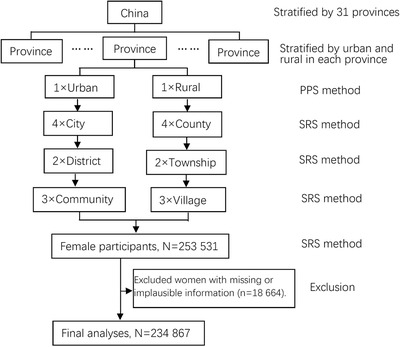
A schematic overview of the patient inclusion and exclusion. PPS, probability proportional to size; SRS, simple random sampling

All participants in this study were derived from the China Hypertension Survey, which was conducted from October 2012 to December 2016 in China.^4^ All 31 provinces in mainland China were covered with a stratified multistage random sampling method to conduct a national representative sampling survey on the general population aged ≥18 years. A total of 487 353 participants from 262 urban resident cities and rural counties were enrolled, of which 253 531 (52.02%) were women. We excluded women with missing or implausible information (*n* = 18 664), and 234 867 women were included in the final analysis (Figure 1). The first stage of sampling was to select four cities in urban areas and four counties in rural areas by using the probability proportional to size method within each province. Then, using a simple random sampling method, two districts or two townships were selected in each city or county, and three communities or villages were selected in each district or township. At the final stage of sampling, a certain number of participants from 14 sex/age groups (men and women aged 15–24, 25–34, 35–44, 45–54, 55–64, 65–74, and ≥75 years) were selected from communities or villages using lists compiled by the local government registers of households.

Informed consent forms were written by all participants, and the study was approved by the Ethics Committee of Fuwai Cardiovascular Hospital (Beijing, China).

### 2.2 Data collection

All patients were interviewed by trained staff using standard questionnaires developed by Fuwai Hospital, which covered social‐demographic characteristics, lifestyle factors, history of diseases, and reproductive factors. The physical examinations were conducted by trained physicians and nurses. Height was measured without shoes using a fixed measuring tape and a standard right‐angle device (accurate to 0.1 cm). Waist circumference was measured at the midpoint of the line between the upper edge of ilium and the lower edge of the 12th rib (to the nearest 0.1 cm). Body weight without heavy clothing was measured using an OMRON body fat and weight measurement device.

### 2.3 Blood pressure measurement

Blood pressure was measured three times by trained medical staff on the right upper arm after the participant was sitting at rest for 5 min, with a 30‐s interval between each measurement with an observer present, using an OMRON HBP‐1300 Professional Portable Blood Pressure Monitor (OMRON, Kyoto, Japan). The average of the three measurements was used for the analysis.

### 2.4 Definitions

According to the Chinese guidelines for hypertension management,^20^ hypertension was defined as systolic blood pressure (SBP) of at least 140 mm Hg, and/or diastolic blood pressure (DBP) of at least 90 mm Hg, and/or use of antihypertensive medication within 2 weeks. Body mass index (BMI) was calculated as weight divided by the square of height (kg/m^2^). Overweight and obesity were defined as BMI between 24.0 and 27.9 kg/m^2^ and BMI ≥28.0 kg/m^2^, respectively. Current alcohol drinking was defined as consuming alcoholic beverage at least once per week in the past month. Current smoking was defined as people who smoked at least 20 packs of cigarettes and was still smoking.

### 2.5 Statistical analysis

Baseline characteristics of participants were presented as mean and standard deviation (SD) for normally distributed data or as a proportion for categorical data. Categorical variables were analyzed using the chi‐square test, and continuous variables were analyzed by one‐way analysis of variance (ANOVA). Spearman correlation analysis was used to explore the relationship between age at menarche and BMI, waist circumference, educational level, and other characteristics. Logistic regression was used to estimate odds ratios (ORs) and 95% confidence intervals (CIs) for hypertension associated with age at menarche (classified as ≤13, 14, 15, 16, and ≥17 years) with 15 years as the reference group. This study further investigated the associations by additionally adjusting for age at recruitment (continuous), BMI (continuous), waist circumference (continuous), region (urban or rural), ethnicity (Han or other ethnicities), education level (elementary or below, junior high school, high school or above), current smoking (yes or no), current alcohol drinking (yes or no), ever pregnant (yes or no), menopause status (yes or no), contraceptive use status (yes or no), breastfeeding experience (continuous), stroke (yes or no), myocardial infarction (yes or no), and family history of hypertension (yes or no). We also examined the risk of hypertension by age at menarche in subgroups of women defined by age at recruitment, education level, and BMI. The threshold of statistical significance was set at *p* < .05. We used R 3.6.2 software to conduct our analyses.

## 3 RESULTS

The characteristics of the study participants are listed in Table 1. The mean age at menarche was 14.8 years, the mean (SD) age of study participants at recruitment was 46.1 (18.9) years, the mean (SD) BMI, SBP, and DBP were 23.5 (3.8) kg/m^2^, 125.6 (20.0) mm Hg, and 74.1 (10.3) mm Hg, respectively. The proportions of women with menarche at age ≤13, 14, 15, 16, and ≥17 years were 25.8%, 23.3%, 18.9%, 13.7%, and 18.3%, respectively. A minority of women were current smokers (1.7%), current alcohol drinkers (6.2%), and users of contraceptives (6.0%). Women who were older at menarche were more likely to have higher BMI, larger waist circumference, primary education, less physical activity, reside in rural areas, have more children, later menopause, and have higher mean blood pressure (*p *< .05). Most women had been pregnant (82.1%) and had breastfed (78.7%).

**TABLE 1:** Characteristics of study participants by age at menarche

	Age at menarche						
Characteristics	≤13 (Mean = 12.7)	14	15	16	≥17 (Mean = 17.8)	All (Mean = 14.8)	*p* value
Number (%)	60608 (25.8)	54727 (23.3)	44274 (18.9)	32283 (13.7)	42975 (18.3)	234867 (100.0)	.037
Age at recruitment, mean (SD)	37.1 (17.2)	41.7 (18.0)	46.7 (18.4)	51.8 (17.1)	59.5 (14.4)	46.1 (18.9)	.000
*Body measurements and lifestyle*							
Body mass index (kg/m^2^), mean (SD)	23.1 (3.8)	23.2 (3.7)	23.5 (3.8)	23.9 (3.8)	24.1 (3.8)	23.5 (3.8)	.000
Waist circumference (cm), mean (SD)	77.8 (10.0)	78.6 (10.0)	79.7 (10.2)	81.1 (10.3)	82.2 (10.4)	79.6 (10.3)	.000
Han ethnicity, *n* (%)	52671 (86.9)	47049 (86.0)	37448 (84.6)	27881 (86.4)	37883 (88.2)	202932 (86.4)	.188
Urban resident, *n* (%)	34824 (57.5)	27165 (49.6)	19604 (44.3)	13677 (42.4)	17265 (40.2)	112535 (47.9)	.037
Education level, *n* (%)							
Elementary or below	5780 (9.5)	7217 (13.2)	8727 (19.7)	8173 (25.3)	16472 (38.3)	46369 (19.7)	.037
Junior high school	26637 (43.9)	27703 (50.6)	23935 (54.1)	18344 (56.8)	22421 (52.2)	119040 (50.7)	.104
High school or above	28191 (46.5)	19807 (36.2)	11612 (26.2)	5766 (17.9)	4082 (9.5)	69458 (29.6)	.000
Current alcohol drinking, *n* (%)	3866 (6.4)	2918 (5.3)	2458 (5.6)	2156 (6.7)	3130 (7.3)	14528 (6.2)	.505
Current smoking, *n* (%)	513 (0.8)	619 (1.1)	760 (1.7)	699 (2.2)	1338 (3.1)	3929 (1.7)	.037
*Reproductive characteristics*							
Ever pregnant, *n* (%)	41554 (68.6)	42709 (78.0)	37504 (84.7)	29708 (92.0)	41303 (96.1)	192778 (82.1)	.285
Ever menopause, *n* (%)	12813 (21.1)	15861 (29.0)	17655 (39.9)	16781 (52.0)	31238 (72.7)	94348 (40.2)	.037
Ever use of contraceptives, *n* (%)	3704 (6.1)	3044 (5.6)	2577 (5.8)	2138 (6.6)	2730 (6.4)	14193 (6.0)	.188
Breastfeeding experience, *n* (%)	39360 (64.9)	40736 (74.4)	35992 (81.3)	28685 (88.9)	40113 (93.3)	184886 (78.7)	.747
*Blood pressure*							
Hypertension, *n* (%)	8859 (14.6)	10310 (18.8)	11329 (25.6)	10523 (32.6)	18831 (43.8)	59852 (25.5)	.037
SBP (mm Hg), mean (SD)	120.2 (16.8)	122.6 (18.1)	125.7 (19.7)	128.9 (21.0)	134.4 (22.2)	125.6 (20.0)	.000
DBP (mm Hg), mean (SD)	72.6 (9.7)	73.6 (9.9)	74.4 (10.3)	75.0 (10.8)	75.6 (11.0)	74.1 (10.3)	.000
Family history of hypertension, *n* (%)	17977 (29.7)	14996 (27.4)	12473 (28.2)	9563 (29.6)	12834 (29.9)	67843 (28.9)	.188
*Other diseases*							
Stroke, *n* (%)	287 (0.5)	308 (0.6)	409 (0.9)	387 (1.2)	865 (2.0)	2256 (1.0)	.037
Myocardial infarction, *n* (%)	95 (0.2)	93 (0.2)	98 (0.2)	116 (0.4)	200 (0.5)	602 (0.3)	.030

Percentages were calculated based on women with complete information for that specific variable.

*Abbreviation*: SD, standard deviation.

**FIGURE 2:**
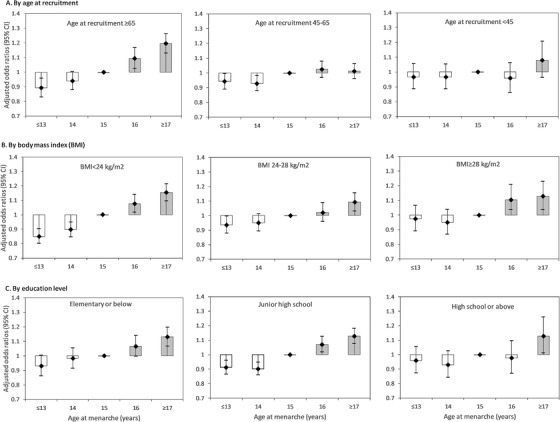
Adjusted odds ratio (OR) (95% confidence interval [CI]) for hypertension by age at menarche. ORs were estimated using a logistic regression model and adjusted for age at recruitment, body mass index, waist circumference, region, ethnicity, education level, smoking, alcohol drinking, family history of hypertension, stroke, myocardial infarction, pregnant, menopause status, contraceptive use status, and breastfeeding experience. Women who experienced menarche at 15 years of age were used as the reference category

**FIGURE 3:**
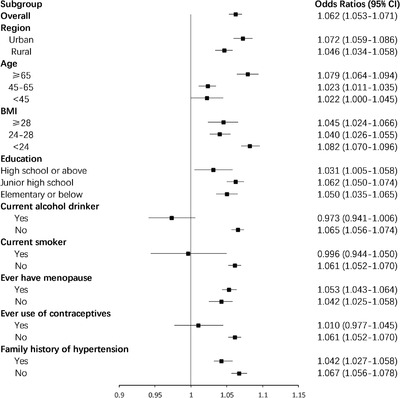
Subgroup analyses of the associations between age at menarche and risk of hypertension according to possible influencing factors. Analyses were adjusted for age at recruitment, body mass index, waist circumference, region, ethnicity, education level, smoking, alcohol drinking, family history of hypertension, stroke, myocardial infarction, pregnant, menopause status, contraceptive use status, and breastfeeding experience

Table 2 shows the adjusted ORs for hypertension by age at menarche (≤13, 14–16, and ≥17 years). After adjustment for age at recruitment, BMI, waist circumference, region, ethnicity, education level, smoking, alcohol drinking, family history of hypertension, stroke, myocardial infarction, and reproductive factors, the ORs (95%CIs) were 0.912 (0.877–0.948), 0.927 (0.893–0.963), 1.000 (reference), 1.061 (1.020–1.102), and 1.129 (1.090–1.169) for those with age at menarche ≤13, 14, 15 (reference), 16, and ≥17 years, respectively. The highest risk was seen in those with menarche at age ≥17 years. After adjustments, ORs (95%CI) of hypertension by age at menarche in subgroups of women defined by age at recruitment, education level, and BMI are shown in Figure 2. We also conducted subgroup analyses according to region, alcohol consumption status, smoking status, age at recruitment, BMI, education level, menopausal status, use of contraceptives, and family history of hypertension (Figure 3). Age at menarche was positively associated with hypertension, with an adjusted OR of 1.062 (95%CI: 1.053–1.071) per year. Positive associations between age at menarche and risk of hypertension were evident among the old, middle‐aged, and young women; among thin, overweight, and obese women; and among low, intermediate, and high education level groups. This association was stronger in urban women and postmenopausal women.

**TABLE 2:** Odds ratio (OR) and 95% confidence interval (CI) for hypertension according to age at menarche

	≤13	14	15	16	≥17
Model 1	0.883 (0.852–0.914)	0.893 (0.863–0.924)	1.000 (reference)	1.108 (1.070–1.148)	1.185 (1.148–1.224)
Model 2	0.907 (0.873–0.941)	0.924 (0.890–0.958)	1.000 (reference)	1.068 (1.028–1.110)	1.158 (1.119–1.198)
Model 3	0.898 (0.865–0.931)	0.906 (0.875–0.939)	1.000 (reference)	1.091 (1.052–1.131)	1.144 (1.107–1.182)
Model 4	0.912 (0.877–0.948)	0.927 (0.893–0.963)	1.000 (reference)	1.061 (1.020–1.102)	1.129 (1.090–1.169)

Model 1, adjusted for age at recruitment.

Model 2, adjusted for age at recruitment, body mass index, waist circumference, region, ethnicity, education level, smoking, alcohol drinking, family history of hypertension, stroke, myocardial infarction.

Model 3, adjusted for age at recruitment, pregnant, menopause status, contraceptive use status, breastfeeding experience.

Model 4, adjusted for age at recruitment, body mass index, waist circumference, region, ethnicity, education level, smoking, alcohol drinking, family history of hypertension, stroke, myocardial infarction, pregnant, menopause status, contraceptive use status, and breastfeeding experience.

## 4 DISCUSSION

In this study, which was nationally representative, the mean age at menarche of the 234 867 women was 14.8 years. We found that women with a history of late menarche had a higher risk of hypertension. The risk of hypertension increased by 6.2% for every 1‐year increase in age at menarche. This association between hypertension and age at menarche also appeared to be similar among subgroups. Our study is the first to report the association between age at menarche and hypertension in Chinese adult women on a national scale and to explore it in several different subgroups.

Although several studies have explored the association between the risk of hypertension and age at menarche in women in China, these studies were limited to one city, one province, or only rural areas, included a smaller number of participants, and the conclusions of those studies were inconsistent. Li^21^ found that women with a history of early menarche had a higher risk of high blood pressure, whereas the participants in this study were all rural postmenopausal women from Henan province in China (*n* = 15 361). Shen^22^ found that age at menarche was not associated with hypertension among urban women aged 45 years and older (*n* = 7893). However, our study suggested that age at menarche positively correlated with the risk of hypertension in Chinese urban women. Han Lei^16^ reported that late menarche tended to be associated with a high risk of hypertension in rural areas of Chongqing City, China.

The relationships between age at menarche and hypertension are also contradictory in other countries. Evidence from 12 336 participants in the Korean National Health and Nutrition Examination Survey showed no significant association between age at menarche and hypertension after adjusting for potential confounders.^23^ However, a study in Brazil^13^ of 33 715 participants found that age at menarche was inversely associated with increased blood pressure.

These inconsistent findings regarding the association between age at menarche and hypertension might result from differences in study design, sample size, race, categories of age at menarche, and different genetic backgrounds between study populations.^17^, ^18^ Participants from different regions have different lifestyle behaviors, living environments, and different confounders, which may lead to inconsistencies in results. In addition, few previous studies have adjusted for age at recruitment, BMI, education level, menopause status and other confounding factors, perhaps resulting in a statistical bias.

Mechanism studies may explain the positively correlation between menarche age and risk of hypertension. Studies have shown that late menarche is associated with low estrogen levels.^24^, ^25^ Estrogen can protect women from cardiovascular diseases and reduce blood pressure by stimulating endothelial nitric oxide synthase.^26^, ^27^ Women who were younger at menarche are more likely to have high ovarian hormone levels, and ovarian hormones can protect women from hypertension and atherosclerotic cardiovascular disease.^28^ In addition, women with late menarche had lower levels of growth hormone,^29^ which is associated with lower blood pressure and low‐density lipoprotein cholesterol.^30^

We found that women with late menarche were more likely to have higher BMI, larger waistlines, smoking, primary education (*p* < .05). These are also possible explanations for our finding that older age at menarche was associated with a higher risk of hypertension. Interestingly, we also found that the mean age at recruitment increased with age at menarche (*p* < .05). The reason for this phenomenon may be that with the improvement of economic and nutritional levels, women are more likely to be precocious. A significant decline in age at menarche has been found in women in China and other countries in recent decades.^31^, ^32^, ^33^, ^34^

Our study has several strengths. First, we are the first to explore this problem with a large representative sample in China, and our patients were adult Chinese women aged ≥18 years. We also conducted a detailed subgroup analysis. We believe that our study reflects the actual correlation between the age at menarche and hypertension in Chinese women. Second, strict quality control measures were taken to ensure data quality and reliability. Third, we adjusted for potential risk factors for hypertension to improve the reliability of our results. There are some limitations to our study. First, the age at menarche was self‐reported and not based on medical documentation. Nevertheless, some studies have shown a high correlation between recalled and actual age at menarche.^8^, ^35^, ^36^ Self‐reporting is an effective method for measuring age at menarche in women.^37^ Second, the cross‐sectional design could not prove a causal relationship.

## 5 CONCLUSIONS

We concluded that older age at menarche was significantly associated with an increased risk of hypertension among adult Chinese women. Each 1‐year delay in menarche was associated with a 6.2% increase in the prevalence of hypertension. This association between hypertension and age at menarche appeared similar among old, middle‐aged, and young women; among thin, overweight, and obese women; and among women with low, intermediate, and high education levels. These findings indicate that knowledge of age at menarche could be used to assess the risk of hypertension in women, thus, enabling them to benefit from early preventive interventions.

## CONFLICT OF INTEREST

The authors of this paper indicated no competing interest.

## AUTHOR CONTRIBUTIONS

Zengwu Wang, Runlin Gao: conceived and designed the study. Lu Chen: analyzed the data and drafted the manuscript. Linfeng Zhang, Zuo Chen, Xin Wang, Congyi Zheng, Yuting Kang, Haoqi Zhou: collected and interpreted the data. Zengwu Wang, Lu Chen: critically revised the manuscript.
